# Veterinarians as Sanitarians1Read before the New York State Veterinary Medical Association, September 8, 1896.

**Published:** 1897-01

**Authors:** Claude D. Morris

**Affiliations:** Pawling, N. Y.


THE JOURNAL
OF
COMPARATIVE MEDICINE AND
VETERINARY ARCHIVES.

VOL. XVIII. *JANUARY*, 1897. No. 1.

### VETERINARIANS AS SANITARIANS.[1]

BY CLAUDE D. MORRIS,
PAWLING, N. Y.

"TIME and experience test the works of man, and the highway of progress is covered with the fragments of countless inventions. The creeds, the dogmas, the social relations of one age become the bywords or the antique curiosities of the next. Men do what they can, and coming generations pardon their errors, but judge their work as they ought. What is good lives, what is bad dies. This is the general rule." We are living in an age that demands of every man the best fruits of his mental and physical energies to meet the exigency of the times. The closing years of the nineteenth century are the ripening stage of the greatest minds that have ever lived—the abundant fruition of the strongest hand that holds the pen or the mechanic's tool. As the months roll by responsibilities multiply upon the shoulders of those who think and work the hardest in the ranks of the professional, architectural, and scientific classes. The struggle is to reach the high peak of human endeavor. Never before have such auspicious opportunities for usefulness and preferment among its members been laid upon the threshold of our profession as lies there to-day. The man of ability and thoughtfulness readily appreciates the situation who with willing hands grasps the rough material fresh from the sources of inexhaustible supply in the professional field, to mould and to polish the roughened stone into a character of usefulness, beauty, and perpetuity. A profession that within a short time has been looked upon with an air of disdain and ridicule by men in other professions is now beginning to be recognized and accepted as a factor beneficent in

[1] Read before the New York State Veterinary Medical Association, September 8, 1896.

165151

the great problem of social economy. Thus it is that which has been accepted in the past as the acme in the science has served its purpose well, but the onward tread of the wheels of progress refuses to accept as an integral the skill of the past; but, on the other hand, is demanding at the hands of the learned the last deductions of the best products of their work. Civilization is gaining friends hourly and enlarging its borders daily. It is utilizing forces that heretofore were latent; that which in the past was refused is now material of usefulness and adornment. As individual members of the veterinary profession we find ourselves to-day in the midst of momentous duties. Responsibilities which are not altogether new, but are forced to the front, demanding a solution as one of the problems incident in our Commonwealth. The question of sanitary science and its practical application by the profession is confronting us with irresistible force. In no small degree are the health and wealth of the Empire State dependent upon our ability and energy. Are we equal to the emergency? The results of our professional career can best answer the question. The consumers of animal flesh and of dairy products are largely at the mercy of the tradesman, with the exception of a few firms, whose sense of honor and justice in their business relations with the public is based on the broad principle of dealing with others as they should like to be dealt with. It is to this class of business men and to veterinarians that the millions in our State who daily consume flesh and dairy products can only depend upon as the proper persons to protect their table from unwholesome foods. The competition in the manufacture and sale of food-products is exceedingly spirited these days. This rivalry does not always tend to increase the merit of the article. The spread of disease among animals themselves, the many avenues by which disease is transmitted from the lower animals to mankind, and its eradication, are questions all of which require a solution at our hands.

There is no one article of food so universal in use as that of milk. Its composition possesses all the elements necessary for primary growth; it is the initial and only food for the newborn, and continues as such for months. One of its physical characteristics is its susceptibility to untoward influences. It will receive and convey the odor of its surroundings. The unpleasant flavor of certain vegetables, sour or fermentative foods which the cow may eat, are readily detected in the milk; and of all food that reaches our table none possesses a more genial habitation for nearly all forms of bacteria as milk. Not a year passes but that we have living proof of the communicability of disease from various sources through the medium of milk. Diseases that are not wholly infantile, but attack all ages in man. Not only has it been proven that certain diseases incident to the bovine family have been carried through milk to man, but that diseases wholly allied to man have been transmitted through milk. Thus this medium is open to a double source of infectiousness; of the two sources of infection we find that in man lies the greater evil of the two. Aside from bovine phthisis there are but few diseases of frequency in that family considered dangerous and liable to transmission through the products of the dairy, but in man we have a number of infectious diseases which can be transmitted through this medium. Whether tuberculosis in man has its essential source in the bovine family or not, there are, however, certain conditions of the disease in the animal which seem even on the surface to be inimical to health, and when considered upon the ground of its possible relation and potency toward creating the disease in man there is at once of itself a wall of defence very difficult to destroy. The Massachusetts Society for Promoting Agriculture, after making 121 microscopical examinations of milk taken from 36 cows on nineteen different occasions, found that the tubercle bacilli actually existed in the milk of 33 per cent. of the animals examined, and that the animals were actually affected with tuberculosis; that the udder was free from the disease, which was proven in all cases by careful post-mortem examinations. The animals selected for this test were known to have the disease, the object of the test being to prove whether or not the bacilli actually existed in milk taken from animals which had the disease in parts other than the udder. On the other hand, we have intimated that by man milk is environed with conditions which lay it open to serious and fatal consequences ere it reaches the consumer's table. The wonder is it is not more so. A daily food so well calculated by constituents to be a suitable abiding-place for germ-life which develops and multiplies indefinitely requires more than ordinary attention while in the hands of the producer and tradesman. A glance at the records, the frequent press accounts of the spread of infectious disease, prove beyond contradiction the value of the assertion. There are many cases on record in which there is undeniable proof of the spread of typhoid fever, diphtheria, and many forms of enteric disease through this medium alone. We beg to cite one prominent case which excited more than local interest, occurring in the village of Stamford, Conn., a year ago last April. Typhoid fever began to appear during the early part of the month. No special significance was attached to its invasion, as at that time but a few cases had developed; but before the month was gone 209 cases had been reported to the health officers. In the meantime diligent search was on foot to find by what means or avenue this intruding enemy was seeking the life and happiness of the community; and before the fell destroyer had been found and from whence it came it had as its claim 386 victims and 22 deaths. By reading a short paragraph clipped from an article on the subject published in a New York paper at the time will explain. It says: "Every one of the cases has been traced directly to a foul well in the stabling-shed of H. R. Blackham, a retail milk-dealer on Greenwich Avenue, in that part of the city known as Waterside. A map was drawn, showing the location of the typhoid cases and Blackham's delivery route. The Stamford doctors have not driven off the milkman's route in their visits. Every house with typhoid patients was a stopping-place for the milkman's horse. Dr. T. Mitchell Prudden analyzed the water from Blackham's well (which was located in the barnyard) and says: 'The number of living bacteria of various kinds in one cubic centimetre is 69,660. This number of living germs would be reasonable in sewer-water or cesspool."'

The dairy industry is a very common one in our country, and especially is this true in the vicinity of our large cities. New York City receives its supply of milk along the lines of railroads for 100 miles north, 250 miles west, and nearly 50 miles south, to say nothing of the smaller cities and villages within this radius which receive their supply also. Of this large territory in the eastern and southeastern portion of the State, nearly all of which is devoted to dairying, but a small portion is under sanitary inspection. In this field I believe we can do a great work for humanity. Impress upon the dairyman the criminality of selling milk from cows which are suffering from disease, and that the act is none the less criminal by selling the animal itself whose flesh ultimately reaches the table of some innocent consumer. A few sanitary principles should be in vogue at the dairy. Thorough cleanliness, light and ventilation of stables, pure water-supply, and wholesome foods are the essentials which should enter into the management of the dairy. We should be in touch with the health officers in our immediate locality, working together in one common cause to maintain the health of the community. This applies more especially to children and the young, whose sensitive tissues and susceptible bodies, if they reach normal maturity, are influenced greatly by the character of the food they receive. How often it is the case that the family physician can trace the enteric trouble in the babe to the milk-can, and not infrequently do we hear of a case of tubercular meningitis in the child of a family whose record is as free from the disease as if the disease never existed, and upon investigation its source is traced to the family cow or the dairy whence they had long purchased their supply. It is along this line of professional work that the veterinarian can make for himself a reputation worthy of his calling. Fellow-practitioner, has it ever occurred to you, when making professional visits among your clients, to make a thorough investigation of the cause of the disease which is under treatment with the same unremitting vigilance with which you treat it; and how multiplied are the instances at the conclusion that the unsanitary surroundings are sufficient cause for the malady? In no other way can we manifest greater professional skill than in being able to remove the cause of the disease rather than by treating it successfully, and especially will this prove true at the dairy farm, whose product is placed on the market for exchange.

Harold Ernst, on the subject of infectiousness of milk, arrives at these conclusions: 1. While the transmission of tuberculosis by milk is probably not the most important means by which the disease is propagated, it is something to be guarded against most carefully. 2. The possibility of milk from tuberculous udders containing the infectious element is undeniable. 3. With the evidence here presented, it is equally undeniable that milk from diseased cows with no appreciable lesion of the udder may and not unfrequently does contain the bacillus of the disease. Mr. President, are we imposing too much on your patience and this assembly by stating that it is our belief that we as veterinarians and sanitarians fall short in our professional duties unless we use all reasonable means to impress upon dairymen who place their product upon the market the necessity of as perfect sanitary conditions as is possible to maintain; and especially, whenever it comes to our notice that milk is offered for sale from cows in an unhealthy condition of body, to use our best endeavor to have them removed from the herd or report the case to the health officer? Neither should milk be sold from dairies in which certain diseases incident to the human family are prevalent and associated with the dairy. Diseases which should bar the dairy product from sale are typhoid fever, diphtheria, pulmonary phthisis, croup, scarlet fever, common measles, German measles, smallpox, erysipelas, and syphilis. There is room for much reform in the dairy sections of our State; and who among the scientists is better qualified to perform so important a public function than the veterinarian.

I will not go into the details of the dairy, which are many and complex, aside from what has already been said as to cleanliness. The method to be pursued and the material used are quite essential. A thorough sweeping down of the walls and ceilings of the stables twice a year, spring and fall, then applying a rich coat of whitewash and carbolic, which is both a disinfectant and parasiticide; the maintaining of water-tight floors and gutters, so that all the excreta can be moved without finding lodgement beneath the floors; with good dainage in the yards; also no wells in the vicinity of the yards; and during the months in which the cows are stabled they should be groomed daily, for the purpose of removing particles of stable-dirt and their own excrement, which is very liable to find its way into the milk-pail, thus becoming a source of contamination. It may seem needless to state that as soon as the milk is drawn from the cow it should be cooled to a temperature of at least 60° F. and kept in an atmosphere free from all odors or contaminating influences of whatever nature until it is ready to be taken to market. We believe that as the result of carelessness and ignorance on the part of some dairymen premature graves have been dug for children; that the bright hope of their maturity, which was twin in the breast of the parent at their birth, has been broken and ran away in bitter tears. If the wealth of a nation lies in the character of the extent of area, in its hidden resource of priceless material and its commerce, then its greatness lies in the character of its human flesh. A strong arm and a sane brain are necessary to lay the foundations of true greatness, and it is in the newly-born babe where these conditions begin.

The question of a final disposition of neat cattle when suffering with disease of a contagious and infectious nature among themselves and capable of transmission to man becomes a matter of vital importance in its relation to public health. The profession should be a unit on this question as best calculated by experience and knowledge to direct the public mind in this very important question, affecting as it does the health and wealth of the nation. What should be done with an actinomycotic animal? Learned men in the profession differ as to the contagious character of the disease, except among bovine animals, and I believe this is disputed by some; the origin of the fungus, if of vegetation, and what relation does it bear to the animal; if by contact through abrasion of the skin or by alimentation, then why not man contract the disease from the same source independently of the animal? However diversified opinion may regard this disease, is it not well to give the doubt the preference? We would not be understood as sentimental, but believe there are occasions when intuitive knowledge or science or, more plainly put, instinct, is to be relied upon when doubt and ignorance abound. What man possessing a cultured sensibility, to say nothing of professional knowledge in medical science, can make himself believe that the flesh of a diseased carcass is a sweet morsel of food? Though it be only localized, receiving its supply of blood from the systemic circulation, giving off its effete material in the form of a ptomaïne, to be partially eliminated through the process of arterialization in the lungs; and then again the remaining portion penetrating to all parts of the body, availing itself of every opportunity for lodgement in the tissues; though attentuated in vital form, it must in time make general the same disease that to outward appearance seems only local. The question of economy should be subordinate to that of health. Therefore, we cannot admit that even thorough cooking is a sufficient guarantee that the meat is properly sterilized. It has been shown by recent investigation that in many diseases it is not the characteristic bacilli of the disease which produce the toxic infection, manifesting the symptoms of the disease, but that it is a subproduct, the result of the dead bodies of bacteria, a ptomaïne, a soluble alkaloid, readily absorbed, capable of producing septic intoxication. Nearly all authorities are agreed that the bacilli present in the blood of tetanic patients are few, and in animals in which the disease was produced artificially the blood was often found sterile, the microbes being found at the seat of the primary infection, and in the tissues between it and the spinal cord, and in the blood itself. We must then conclude that the laboratory from whence the ptomaïnes come are the tissues in which localization takes place; and it differs not whether the disease is local or general if of importance and contagious in its nature, the blood and the tissues possess "the septic intoxication which is carried by the presence of dead tissue in the body in a state of putrefaction, from the presence of putrefactive bacilli, and that the immediate cause of the intoxication is the absorption of pre-formed ptomaïnes from such a local focus of putrefaction."

Are we justified, in the light of present knowledge touching so important a question, to assent by silence to the sale of a food-product contrary to revealed knowledge? Or if, on the other hand, our busy life hinders personal investigation, that we may know from experience these facts, are we to doubt the result of investigation made by others who have investigated? When human life and happiness hinge on any one question too much is at stake to admit of theory, long drawn-out deductions, and hair-splitting conclusions to prove that under such and such conditions certain agencies and elements are harmless. No; better things are expected of us. We are in a position to lead and become the sanitarians of our time.

The transmission of disease by clinics, manifesting itself in typical form in other patients remote from the seat of infection, which, at the time being, are suffering slightly from some form of enzootic disease or are to undergo some ordinary operation, is of sufficient importance to engage attention in our daily practice. Its influence relative to the commercial value of the lower animals is too significant to admit of casual treatment, the operator acting as the medium by which the infection is conveyed. I will cite two cases, one in which the present speaker was the medium of transmission in one case and the other a regular practising physician. In the spring of 1891 we were treating a case of tetanus in a filly two years old. She and her brother, one year old, were running together in an open shed. They were separated by putting a partition through the enclosure. After the filly recovered the owner wanted the horse colt castrated. This was done by the usual method of casting the animal, removing the testicles, with no bad results following. One week later we were called upon to castrate another colt five miles distant from the farm where tetanus had existed, using the same casting rig. In this case I ligatured the spermatic artery with sterilized catgut. The colt apparently did well until about one week later, when the owner reported that something was wrong with the colt and wished I would go out and examine him. As soon as I saw the colt the symptoms presented like an open book portraying the trouble. It was tetanus in all its agony. Without going into details, which I afterward did to prove whence came the germs of the disease, we will conclude the clinic by stating the fact that the bacilli of tetanus were on the ropes and straps of the throwing-rig, and the disease thus conveyed to the wound of the scrotum. After relating this incident to a physician of large experience he unbosomed a like circumstance in his own practice. He was treating a case of peritonitis which was the result of a wound in the lower third of the abdomen, the wound presenting septic symptoms. One day after dressing the wound he was called to a case of midwifery, in a woman enjoying ideal health; delivery occasioned slight lacerations, labor lasting only three hours. The third day he noticed evidences of inflammation in the parts, extending to the mucous membrane of the vagina. Again, without going into details, which he related, the doctor was satisfied that by his hand he had transmitted the germ of sepsis to a healthy person.

Surgical sanitation means more than ordinary cleanliness. It means perfect sterilization of hands and instruments. In a diversified practice this danger is more eminent, as it brings in contact with the medium a greater number of avenues through which to spread infection. A few months ago we were called to see a case that upon examination proved to be glanders, which was under treatment by an empiric who did not recognize the disease. He also had under treatment at the same place the favorite cat, suffering with a lacerated wound of the arm. Strange to say that in nine days this cat had developed the disease sufficiently to make diagnosis easy. Herein lies the danger, and how often it comes to the surface in practice. The patient apparently doing well; soon we observe complications which are hard to account for, they are absent in the literature of the disease; if, then, these symptoms are not the natural sequel, what are they? Experience tends to confirm the belief that too often we are the medium conveying the germs of disease.

The needs in the profession to-day are closer attention to sanitary principles governing the treatment of disease as well as the prevention of disease. Preventive measures should be based upon pathology—the influences of inheritance. The transmission of functional peculiarity associated with this inheritance of soil and the transmission from parent to offspring of certain conditions, apparently pertaining to the tissues and predisposing to disease are questions for concern as vital as the treatment of disease when precipitated. We can no longer cast aside the necessity of the hour. We are in the field of activity; we must perform. It becomes us, therefore, to do our part well, take on the harness of rich opportunity, toil for the benefit of our race, and at the end of labor reap reward.

---

Chloral hydrate and bromide of potash combined in watery solution gives excellent results in controlling epileptiform seizures in dogs that so often arise from intestinal irritation. A generous dose of sweet or castor oil in the interim of attacks is of advantage.

